# Osteoid Osteoma of the Calcaneus and Patella: A Report of Two Cases and Review of the Literature

**DOI:** 10.7759/cureus.102243

**Published:** 2026-01-25

**Authors:** Ioannis Kougioumtzis, Platon S Papageorgiou, Efthymios Iliopoulos, Stylianos Tottas, Nikolaos Andrianos Ververidis, Nikolaos Gravvanis, Athanasios Ververidis

**Affiliations:** 1 Sports Medicine and Orthopaedic Traumatology, Arthroscopy Unit, General Hospital of Nikaia “Agios Panteleimon”, Nikaia, GRC; 2 Orthopaedics and Traumatology, National and Kapodistrian University of Athens, Athens, GRC; 3 Orthopaedics, University General Hospital of Alexandroupolis, Democritus University of Thrace, Alexandroupolis, GRC; 4 Orthopaedics, General Hospital of Serres, Serres, GRC; 5 Orthopaedics, General Hospital of Chalkida, Chalkida, GRC; 6 Sports Medicine, Athens Kolonaki Orthopaedic and Sports Medicine Center (AKOSMC), Athens, GRC; 7 Orthopaedics, Central Clinic of Athens, Athens, GRC

**Keywords:** benign lesion, benign tumors, bone lesion, bone tumors, calcaneus, non-cancerous tumor, non-malignant lesion, osteoid osteoma, patella

## Abstract

The presence of an osteoid osteoma (OO) in the calcaneus and patella remains an uncommon condition. These lesions can often lead to diagnostic and therapeutic difficulties. Initial assessment and cautious management contribute to excellent results and limited complications.

We report a 20-year-old male with OO of the calcaneus and a 35-year-old male with OO of the patella. The clinical suspicion, in combination with thorough imaging control, can lead to the final diagnosis. Imaging examinations usually reveal a nidus; however, computed tomography remains the gold-standard tool for the detection and diagnosis of lesions. The state-of-the-art therapeutic methods emphasize minimally invasive techniques. The unusual location of these tumors emphasizes the specific need for careful management and minimally invasive treatment, which ultimately contributes to excellent outcomes with minimal adverse effects.

## Introduction

Osteoid osteoma (OO) is a usually benign osteoblastic tumor, typically small in size, found mainly in young patients [1-3]. While these tumors are generally well characterized and manageable, they can pose significant diagnostic and treatment challenges, especially when located in rare anatomical sites such as the calcaneus and the patella [4].

OOs are commonly found in long bones, but their incidence in the calcaneus is rare, with only a few reports in the literature [5]. When it comes to the hindfoot, OO can mimic more common pathologies of the area, such as stress fractures [5,6]. Its occurrence in the patella is also rare, accounting for less than 1% of all cases [7]. Patients with OO in the patella typically present with persistent, localized anterior knee pain that worsens at night and is relieved by nonsteroidal anti-inflammatory drugs (NSAIDs) [7]. The diagnosis is often delayed due to its uncommon location, leading to misinterpretation as other patellar pathologies, such as tendonitis, stress fractures, Brodie’s abscess, and osteoblastoma [8].

Imaging assessment, including plain radiographs and computed tomography (CT) scans, is crucial for accurate diagnosis. Often, a characteristic nidus is present within either the patella or the calcaneus bone [9]. Early and accurate diagnosis is essential, as these tumors are highly responsive to minimally invasive treatments such as radiofrequency ablation (RFA), interstitial laser ablation (ILA), and microwave ablation (MWA) [10]. Recently, CT-guided percutaneous techniques (CT-RFA) have gained ground as potential therapeutic options due to their satisfactory outcomes [11,12].

In the present article, we report two cases of OO in the patella and the calcaneus and their treatment. A review of the recent literature is also performed regarding OOs in these rare anatomical locations. The clinical manifestations, therapeutic approaches, and final treatment protocols described in the existing literature are concisely presented. Understanding the specific characteristics of OOs in unusual locations, such as the calcaneus and the patella, is crucial for ensuring immediate diagnosis, effective management, and the prevention of unnecessary complications.

## Case presentation

Case 1

**Figure 1:**
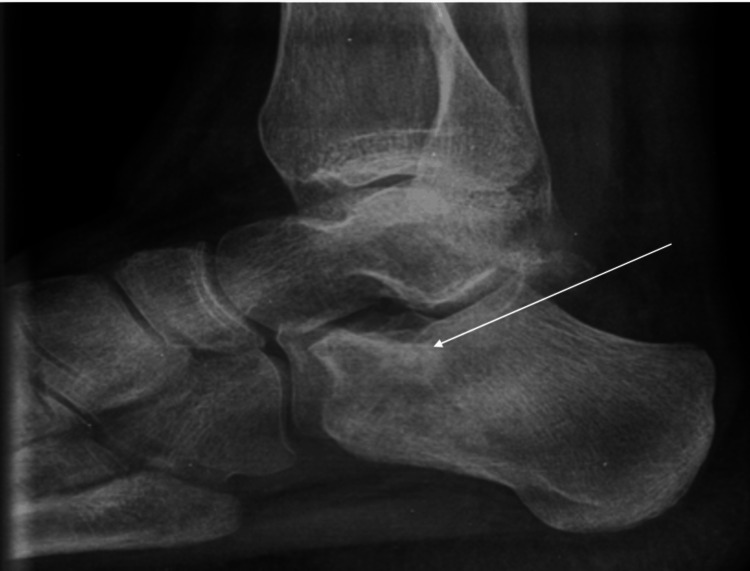
Plain X-ray (sagittal view) of calcaneus The white arrow depicts a small, central periosteal core reaction with cortical thickening (nidus) on the anterior and superior aspect of the calcaneus. This lesion is visible as a radiolucent area.

A 20-year-old male patient with a two-year history of gradually increasing atypical nocturnal pain, located in the right heel, without a history of trauma, presented to the outpatient clinics of our hospital. During clinical examination, atypical calcaneal pain was the only symptom, without any other pathology. Radiological evaluation with anteroposterior, lateral, and skyline plain radiographs of the calcaneus was performed (Figure 1). 

**Figure 2:**
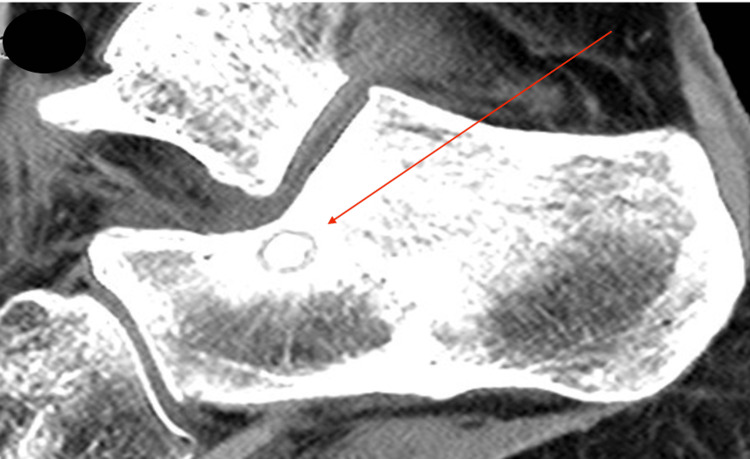
Sagittal view of computed tomography of calcaneus The red arrow depicts a circular area (nidus) surrounded by dense bone in the anterior calcaneal region.

Then, a CT scan was performed, which revealed a nidus in the calcaneus, confirming the diagnosis (Figure 2).

**Figure 3:**
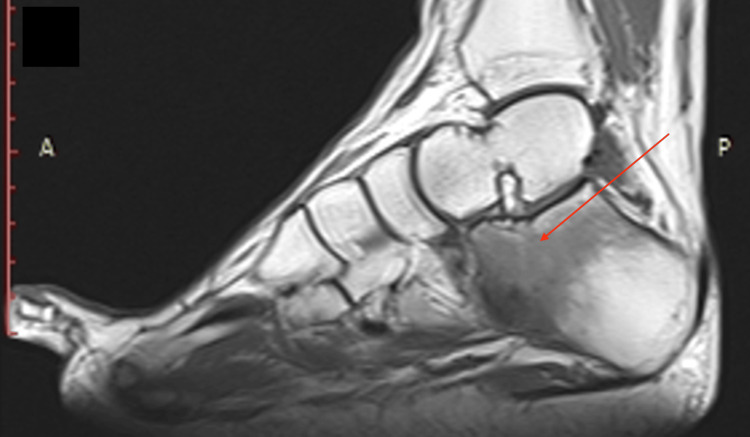
Sagittal view of T1-weighted magnetic resonance imaging (MRI) of calcaneus The red arrow indicates a small, rounded, well-defined nidus and diffuse bone marrow oedema (low signal - dark) in the affected anterior, superior, and middle regions of the calcaneus.

A well-circumscribed border of the nidus, as well as a radiolucent area surrounded by sclerotic bone within the calcaneal bone, was depicted on the CT scan. Additionally, a magnetic resonance imaging (MRI) was performed to further evaluate the surrounding soft tissue and bone marrow abnormalities of the calcaneus and the well-circumscribed lesion (Figure 3).

**Figure 4:**
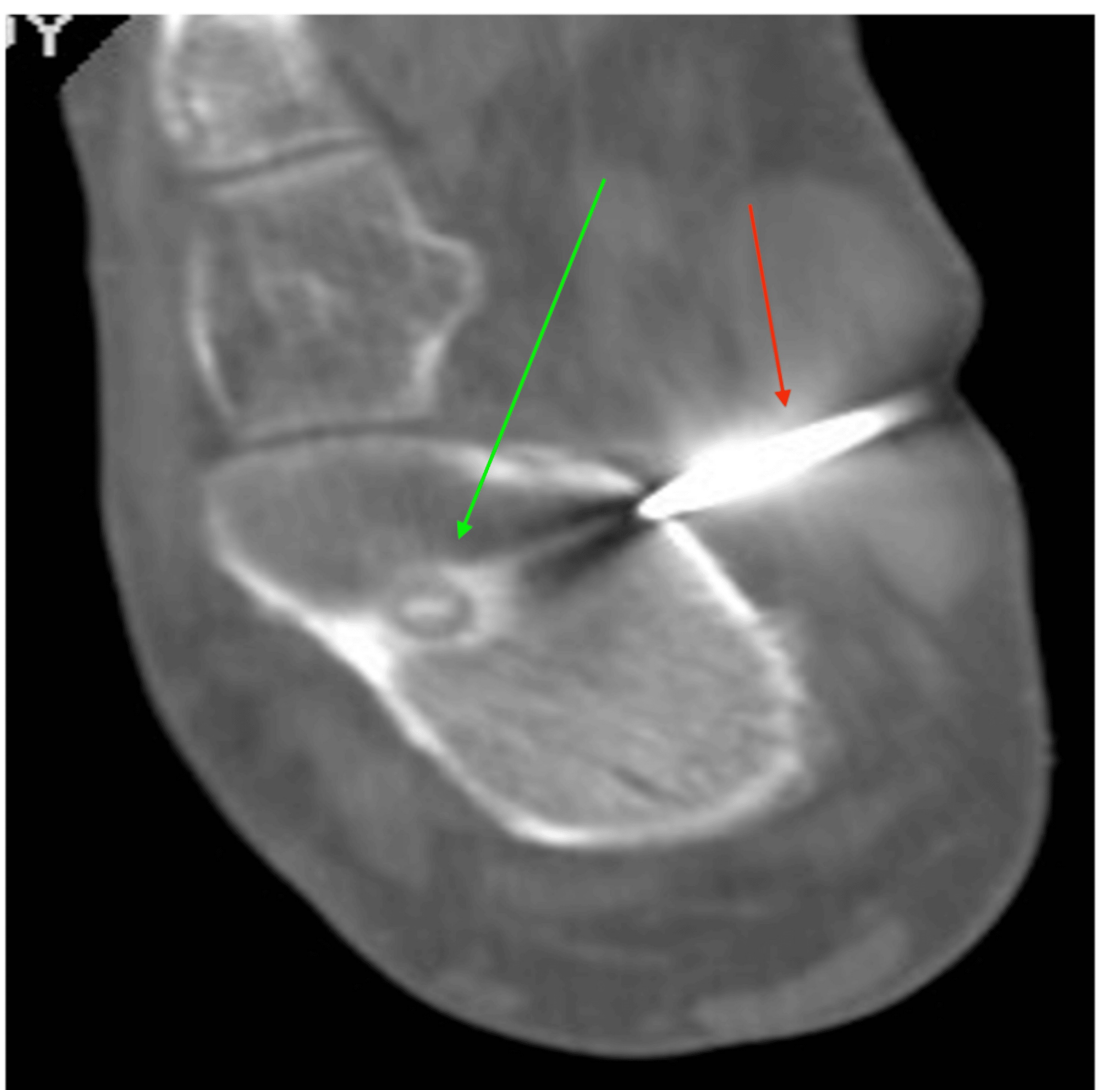
Computed tomography (coronal view)-guided percutaneous radiofrequency ablation (RFA) of the calcaneus The red arrow indicates the needle passing through the skin and soft tissues into the calcaneus. The green arrow points to the well-defined nidus in the anterior and middle regions of the calcaneus.

The patient ultimately underwent CT-guided radiofrequency thermal ablation of the lesion (Figure 4).

The patient was immediately asymptomatic (Visual Analog Scale, VAS: 0). In the first postoperative days, he walked with crutches and was able to bear full weight within one week. Furthermore, within two weeks, no limping was observed, and a full range of motion was evident. The total follow-up was eight years.

Case 2

**Figure 5:**
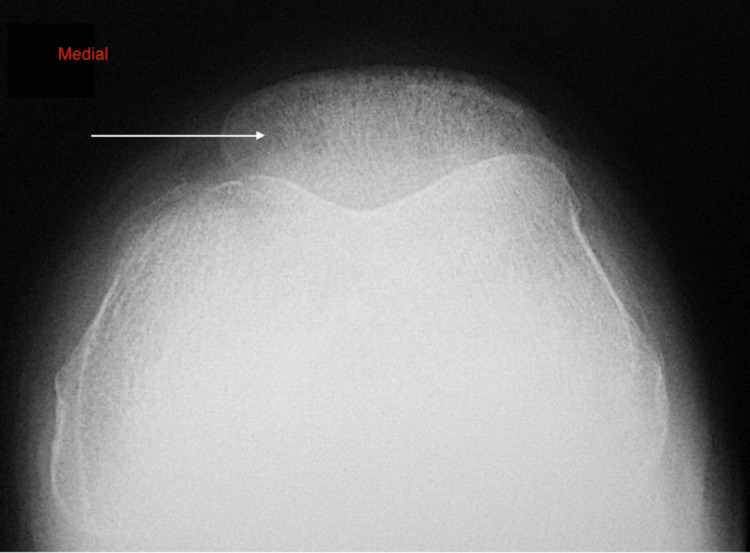
Transversal view of radiological image of the patella The white arrow denotes the nidus in the medial area.

The second case concerns a 35-year-old male patient with chronic anterior left knee pain for about one year. Clinically, the patient was tender over his patella. The rest of the knee examination was normal, with no effusion and a full range of motion. The blood tests, which were performed, did not reveal any pathological values. Plain radiographs did not reveal any obvious pathology (Figure 5).

**Figure 6:**
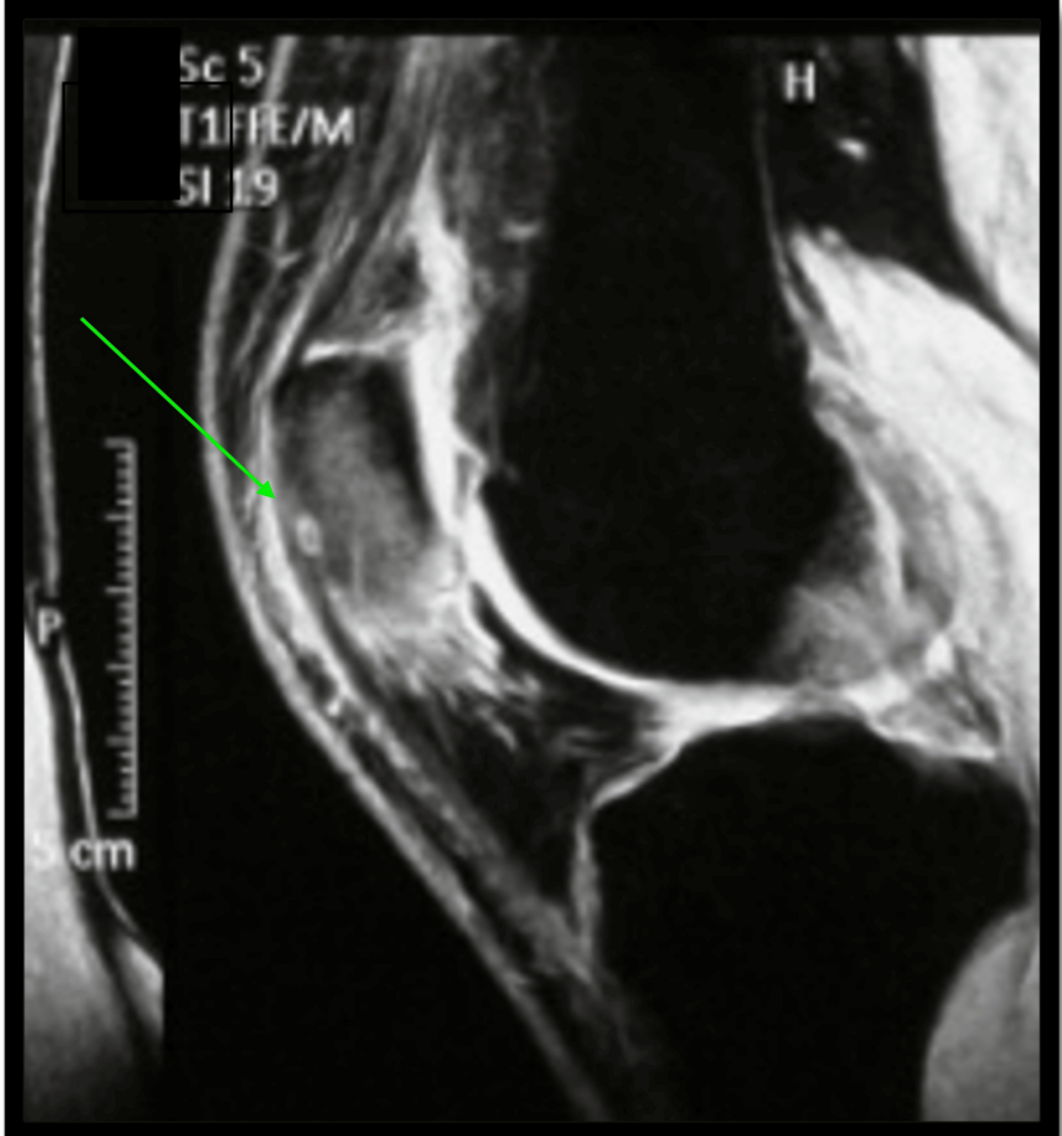
Sagittal view of T2-weighted magnetic resonance imaging (MRI) of the patella Diffuse bone marrow edema (high signal intensity) in the affected patella, and a well-circumscribed periosteal lesion at the inferior pole of the patella (green arrow).

MRI of the knee illustrated a pattern of diffuse bone marrow edema throughout the patella, and a well-circumscribed periosteal lesion at the inferior pole of the patella (Figure 6). 

The patient underwent arthroscopic assessment and an open thermal ablation of the lesion. The knee joint arthroscopy did not reveal any other pathology. The patient was asymptomatic and fully functional at the eight-year follow-up. 

## Discussion

OO is a benign osteoblastic lesion that constitutes approximately 11% of all benign bone tumors and 3% of all primary bone tumors [7,13,14]. It can be classified as intra-cortical, subcortical, or intramedullary [5,7,13]. The main characteristic is the small nidus (less than 15 mm in diameter), surrounded by a radiolucent area, which is in turn surrounded by sclerotic bone [8,14,15]. Furthermore, OO is generally observed in children and young adults, twice as often in men, and mostly between the ages of 10 and 25 [16]. In terms of location, it is often detected in the femur and tibia (in over 50% of cases). It can also be found in the fibula, humerus, vertebra, talus, calcaneus, and patella [5,17,18]. OOs in the calcaneus and patella are rare, with the occurrence in the calcaneus comprising 2.7% and in the patella 0.034% of all OOs [7,15]. The rarity of these locations often leads to diagnostic challenges and delays, as the symptoms may mimic other, more common conditions affecting these areas.

The initial assessment commences clinically, with characteristic findings such as pain, worsening at night, and usually relieved by NSAIDs or aspirin [17-19]. Specifically, the nocturnal nature of the pain is one of the hallmarks of this condition, often leading to sleep disturbances and significant discomfort [17,20]. Additionally, clinical signs and symptoms in the calcaneus may include edema, structural deformity, muscular atrophy, and restrictions in mobility [3,5,14,15,21,22]. Non-specific clinical features in calcaneus and patella OOs may lead to delayed diagnosis [16,17,19-21,23]. Therefore, a high index of suspicion from clinicians is necessary to diagnose this condition, which is often challenging [4]. Our review reveals that the initial diagnosis is often incorrect, and the time between symptom onset and final diagnosis ranges from 3 to 60 months. Secondly, the mean time of delayed diagnosis ranged from 14 to 36 months. In our case, calcaneus OO symptoms preceded diagnosis by nearly two years. Pathologies that could be considered in the differential diagnosis include plantar fasciitis, equinus contracture, or stress fractures [5,16,24]. A comparison of the stated and present cases is illustrated in Table 1.

**Table 1:** Cases with an osteoid osteoma of calcaneus Abbreviations: M: male; F: female; NSAIDs: nonsteroidal anti-inflammatory drugs; NR: not reported; m (ms): month (months); d (ds): day (days); y (ys): year (years); RFA: radiofrequency ablation; RA: rheumatoid arthritis; CT: computed tomography; R: right; L: left; w: week; ROM: range of motion

Studies	Cases	Gender	Age	Size	Symptoms	Symptom duration	Initial diagnosis	Intervention	Follow up
Wallace et al. (2016) [13]	1	M	38	12 mm	1. Nocturnal pain, 2. relieved with NSAIDs	8 ms	NR	RFA	56 ds
Jurina et al. (2017) [14]	1	M	19	NR	1. Ankle (pain, swelling), 2. pain worse (night, exercise)	5 ms	Peroneal tenosynovitis	Arthroscopic excision	8 ms
Christodoulou et al. (2003) [15]	1	F	19	NR	1. Pain; 2. swelling; 3. treated with NSAIDs; 4. recurrent (5 ys), 4.1. nocturnal pain, 4.2. untreated with NSAIDs	5 ys	Monoarticular juvenile RA	Surgical excision under fluoroscopic control	1 y
Aratake et al. (2012) [16]	1	F	18	NR	1. Nocturnal pain, 2. swelling, 3. heat sensation and tenderness, 4. recurrent night pain (7 ms postoperative)	5 ms	Inflammatory arthritis	Surgical excision	2 ys
Lo et al. (2012) [3]	1	F	8	Nidus: 5 mm	1. Nocturnal pain, 2. limping, swelling, and tenderness, 3. limited ROM, 4. not improved after NSAIDs	6 ms	Inflammatory arthritis	NR	NR
Daniilidis et al. (2012) [17]	3	NR	NR	NR	Pain (night, rest)	NR	NR	RFA	2 ws
Hamada et al. (2016) [5]	1	M	19	Nidus: 7 mm	1. Persistent pain, 2. swelling, 3. nocturnal pain	6 ms	Stress fracture and subtalar arthritis	Surgical excision	1 y
Tauheed et al. (2016) [18]	1	M	22	NR	NR	NR	NR	Arthroscopic excision	NR
Migues et al. (2005) [19]	1	M	39	NR	1. Persistent pain, 2. swelling, 3. relieved with NSAIDs	8 ms	NR	Percutaneous RFA (under CT guidance)	3 ys
Papachristos and Michelarakis (2016) [20]	3	M	10	NR	Nocturnal pain	19 ms	Subtalar synovitis	Surgical excision	>3 ys
		F	12	NR	1. Hip pain, 2. nocturnal pain	17 ms	Hip pain		
		F	11	NR	Pain relieved with NSAIDs	21 ms	Equinus contracture		
Morris and Goldman (2003) [21]	1	M	11	NR	1. Limping, 2. pain (exercise)	6 ms	NR	Surgical excision	23 ms
Sanhudo (2006) [22]	1	M	30	6 mm	1. Nocturnal pain, 2. limited ankle plantarflexion	4 ys	OS trigonum syndrome	2 operations (recurrent after 1st operation)	4.8 ys
Tsukada et al. (2020) [23]	1	M	17	NR	1. Nocturnal pain, 2. restricted ROM, 3. slight swelling, 4. pain decreased with NSAIDs	6 ms	NR	Arthroscopic excision	2 ys
Kostrzewa et al. (2014) [24]	1	M	18	4 mm	Dull pain	>1 y	NR	Microwave thermal ablation (using dynamic MRI)	6 ms (1 w: symptom free)
Okuda et al. (2003) [25]	1	M	30	15 mm	1. Pain (walking, rest), 2. spasm tibialis anterior muscle, 3. swelling and tenderness, 4. restricted ROM	>1.5 ys	Ankle sprain	Surgical excision	46 ms
Yanget al. (2011) [26]	1	M	24	Nidus: 4 mm	1. Pain, 2. swelling, 3. bruising	3 ms	1. Avulsion injury calcaneo-fibular ligament, 2. calcaneal stress fracture	Surgical excision	NR
Payo-Ollero et al. (2021) [27]	6	M	20	NR	Nocturnal pain	12 ms	NR	Surgical excision (curettage and bone crafting)	46, 1 ys
		M	13	NR		19 ms	NR	Surgical excision (curettage and bone crafting)	39, 4 ys
		F	11	NR		27 ms	Chronic synovitis	Surgical excision (curettage and bone crafting)	35, 7 ys
		M	13	Nidus: 4.82 mm		12 ms	Ankle sprain	Surgical excision (CT-guided)	21, 8 ys
		F	16	Nidus: 4.78 mm		4 ms	Ankle instability	Surgical excision (CT-guided)	16, 7 ys
		M	47	NR		4 ms	NR	Surgical excision (curettage and bone crafting)	4 ys
Peyser et al. (2009) [28]	2	NR	NR	NR	1. Nocturnal pain, 2. improved with NSAIDs	NR	NR	CT-guided RFA using water-cooled probe	NR
Peyser et al. (2007) [29]	1	NR	NR	NR	Pain	NR	NR	CT-guided RFA using water-cooled probe	2 ys
Shukla et al. (2010) [30]	4	M	25	Nidus: 4 mm	NR				
		M	25	Nidus: 6 mm					
		M	20	Nidus: 6 mm					
		M	15	Nidus: 10 mm					
Woertler et al. (2001) [31]	1	NR	NR	NR	1. Pain (local), 2. pain worse (night), 3. relieved with NSAIDs	NR	NR	CT-guided RFA	NR
Ren et al. (2017) [32]	1	F	17	NR	Nocturnal pain	2 ys	Chronic osteomyelitis	Surgical excision + autogenous bone grafting + subtalar arthrodesis	2 ys
Gürkan and Erdogan (2018) [33]	3	M	16	NR	1. Pain (nocturnal), 2. pain (weight bearing), 3. tenderness, 4. response to pain relievers, 5. antalgic gait	12 ms	NR	RFA	>2 ys
		M	30	NR		36 ms		Cortical peeling	
		M	15	NR		18 ms		Surgical excision	
Cuesta et al. (2018) [34]	11	NR	NR	13 mm nidus (n: 1)	Nocturnal pain	NR	NR	RFA (skin cooling techniques)	NR
Ζouari et al. (2008) [35]	1	M	16	13 mm	NR	12 ms	NR	CT-guided percutaneous laser photocoagulation therapy	49 ms
Rolvien et al. (2016) [36]	1	M	22	4.5 mm	Severe pain	3 ys	NR	Surgical excision	6 ws
Ayas et al. (2020) [37]	2	M	14	NR	Pain	NR	NR	Surgical excision	>1 y
		F	27	NR					>1 y
Martel et al. (2005) [38]	3	F	12	NR	NR	>12 ms	NR	RFA with 1 cm exposed cool-tip electrode	No recurrence
		F	5	NR					
		M	26	NR					
Etienne et al. (2013) [39]	1	M	18	10 mm	Pain	NR	NR	Interstitial laser photocoagulation	45 ms
Alharbi et al. (2023) [6]	1	F	21	NR	Pain	3 ys	NR	Radiofrequency ablation	18 ms
Ngo and Cao (2023) [40]	1	M	39	NR	Pain	20 ms	Narrowing of the anterior ankle chamber	Surgical excision	5 ms

Concerning the patella OOs, these are often confused with chondromalacia patella [17,19,25]. The delayed diagnosis could extend from 2 to 36 months. In our case, the patient presented with chronic knee pain one year before the definitive diagnosis. Finally, it is worth noting that symptoms are not often relieved with NSAIDs or acetylsalicylic acid [3,7,8,23]. A current review of the literature is displayed in Table 2.

**Table 2:** Cases with an osteoid osteoma of patella Abbreviations: M: male; F: female; y: years; CT: computed tomography; m (ms): months (months); y (ys): year (years); NSAIDs: nonsteroidal anti-inflammatory drugs; NR: not reported

Study	Case	Gender	Age	Size	Symptoms	Duration of symptoms	Initial diagnosis/differential diagnosis	Intervention	Follow-up
Altinel et al. (2007) [41]	1	F	9	3 mm	1. Anterior knee pain, 2. pain (night), 3. response to NSAIDs	3 ys	Chondromalacia patella	CT-guided percutaneous drilling	2 ys
Bavaneh et al. (2018) [42]	3	M	26	7 mm	Knee pain	1 y	Chondromalacia patella	Surgical resection and mosaicplasty	48 ms
		M	21	6 mm	1. Knee pain (night), 2. response to NSAIDs	29 ms	NR		29 ms
		F	49	NR	1. Pain (knee, patellofemoral joint), 2. response to NSAIDs	2 ms	NR		16 ms
Chillemi et al. (2013) [43]	1	F	13	8 mm	1. Anterior knee pain, 2. pain (night)	6 ms	Patellofemoral malalignment	CT-guided percutaneous drilling	2 ys
Georgoulis et al. (2002) [44]	1	F	13	NR	Anterior knee pain	NR	Chondromalacia patella	NR	NR
Ma et al. (2011) [45]	2	M	14	7, 5 mm	1. Knee pain, 2. swelling, 3. pain (night)	1 y	Previous trauma	Surgical resection (curettage operation)	1 y
		M	29	5 mm	1. Knee aching pain 2. pain (night)	2 ys	NR	Surgical resection (curettage operation)	2 ys
Sharma et al. (2020) [7]	1	M	16	7 mm	1. Anterior knee pain, 2. pain (night), 3. modest response to NSAIDs	2 ys	NR	CT-guided radiofrequency ablation	14 ms
Etienne et al. (2013) [39]	2	F	24	4 mm	Predominantly nocturnal pain	NR	NR	Interstitial laser photocoagulation	36 ms
		M	16	6 mm		NR	NR		15 ms
Franceschi et al. (2008) [46]	1	F	16	8 mm	1. Anterior knee pain, 2. pain (night), 3. response to NSAIDs	NR	Patellofemoral dysplasia	CT-guided, arthroscopic-controlled en bloc excision and autologous bone graft	3 ys
Papagrigorakis et al. (2019) [8]	1	M	17	NR	1. Anterior knee pain, 2. pain (night), 3. response to NSAIDs	1 y	Sinding-Larsen-Johansson syndrome, Hoffa’s syndrome	CT-guided radiofrequency ablation	2 ys
Vallianatos et al. (2006) [47]	1	F	51	9 mm	1. Knee pain (intense, aching), 2. pain (day and night), 3. no response to NSAIDs	2.5 ys	Osteochondritis dissecans	Surgical resection	4 ys

The diagnosis is further strengthened by the imaging findings from radiographs, CT, MRI, ultrasound scan, bone scintigraphy, and single-photon emission computed tomography (SPECT) [13,14]. However, the combination of medical imaging methods is often valuable in achieving a definitive diagnosis [7]. Due to the non-specific symptoms, the initial diagnosis is usually made with a plain radiograph [16]. However, X-rays are unable to identify the nidus in 80% of cases [8]. The reason is that the core of the lesion may be small or obscured by the surrounding bone sclerosis. The CT scan is the gold standard method [8,23] that leads to the diagnosis and can detect the distinctive findings of OO, such as the nidus with central calcification and the peripheral sclerotic rim [26]. Additionally, the detailed images provided by CT scans allow for precise localization of the nidus, facilitating better treatment planning. MRI can be used to visualize any surrounding bone marrow edema or other pathology of the surrounding tissues [5], while bone scintigraphy may depict enhanced radionuclide absorption in the affected area [3,20]. Scintigraphy could help highlight areas of increased bone turnover, which is a hallmark of OO, especially in atypical locations [7,27]. In a recent study, Sharma et al. used SPECT/CT to diagnose OO, presenting 100% sensitivity and specificity. This high diagnostic accuracy is particularly useful in difficult-to-diagnose cases, such as those described in the present study. However, more research is needed in this field [7].

The treatment interventions regarding OO can be divided into two broad categories: invasive and minimally invasive. According to Papagrigorakis et al., surgical resection (en bloc and curettage) is widely considered an efficient therapeutic method, since it has been proven to be particularly successful in terms of nidus excision, leading to symptom relief [8]. However, surgical treatment carries the risk of complications, such as fracture, prolonged healing, and even the potential for incomplete resection leading to recurrence [13,15]. Moreover, prolonged hospitalization and rehabilitation periods are additional disadvantages of the surgical method [18,28]. The most prevalent treatment is RFA, reaching a success rate of >90% [29], with limited complications compared to surgical methods [30]. However, Gürkan and Erdogan suggest that RFA should be avoided in cases where neurovascular and ligamentous structures may be at risk [33]. If the nidus is partially ablated, the symptoms may persist [12]. In our cases, RFA was successful, without any recurrences.

High success rates can be achieved with additional minimally invasive interventions. These techniques can provide alternative options for patients who may not be suitable candidates for RFA or surgery [3,15,20,30,31]. A method similar to RFA is MWA [30]. The advantages of this method are remarkable, with no need for cooling techniques or grounding pads, a shorter ablation time, practical application, and a lower risk of skin damage [30]. A supplementary minimally invasive technique is laser photocoagulation treatment, based on thermal destruction of the nidus [32]. Etienne et al. reported favorable results in relation to RFA, due to low cost, low infection risk, and the lack of current flow through the patient’s body. The potential limitation in its application is the expensive equipment [39]. Furthermore, Tauheed et al. first described arthroscopic resection of calcaneal OO, which led to shorter hospitalization and rehabilitation periods and a limited risk of perioperative infection [18]. The arthroscope’s optical magnification allows easy identification of small lesions, which makes this technique especially beneficial for treating lesions in joints and other difficult-to-access structures, such as the patella and calcaneus. On the contrary, these methods, despite early favorable findings, need to be supported by more extensive literature and stronger evidence [15,20,30-32]. 

## Conclusions

The calcaneus and the patella are uncommon sites for OOs. These lesions are characterized by non-specific and unclear clinical findings, which make a high clinical suspicion essential for diagnosis. The diagnostic algorithm requires imaging assessment. CT is the gold standard for diagnosing the pathology; however, additional imaging, such as plain radiographs and MRI, can be valuable. Several therapeutic techniques have been described; nevertheless, a definitive solution with excellent results and limited adverse effects can be achieved with thermal ablation.
